# The Link Between Serum Levels of Dehydroepiandrosterone and Alzheimer’s Disease: A Pilot Study in the Serbian Population

**DOI:** 10.7759/cureus.94489

**Published:** 2025-10-13

**Authors:** Nemanja Nenezic, Jelena Dragicevic Jeremic, Nemanja Rancic, Bratislav Dejanovic, Dejan Kostic, Dragana Nenezic, Teodora Safiye, Christos Alexopoulos, Smiljana Kostic

**Affiliations:** 1 Department of Medical Studies, Academy of Educational and Medical Vocational Studies, Kruševac, SRB; 2 Department of Neurology and Psychiatry, Health Center Knjazevac, Knjazevac, SRB; 3 Center for Clinical Pharmacology and Institute of Radiology, Military Medical Academy, Belgrade, SRB; 4 Institute of Medical Biochemistry, Military Medical Academy, Belgrade, SRB; 5 Institute of Radiology, Military Medical Academy, Belgrade, SRB; 6 Clinic for Otorhinolaryngology and Maxillofacial Surgery, University Clinical Center of Serbia, Belgrade, SRB; 7 Department of Psychology, State University of Novi Pazar, Novi Pazar, SRB; 8 Clinic for Neurology, Military Medical Academy, Belgrade, SRB

**Keywords:** alzheimer's disease (ad), dehydroepiandrosterone sulfate, hydrocortisone, mental cognition, neurosteroids

## Abstract

Background and aim

Alzheimer’s disease (AD) is the most prevalent type of dementia and a major health concern among the elderly population. Considering the role of neurosteroids in neurodegenerative processes, this pilot study aimed to investigate the association between serum dehydroepiandrosterone sulfate (DHEA-S) and cortisol concentrations, as well as their ratio, in patients with AD compared with cognitively intact individuals.

Methods

An observational case-control study was conducted involving 45 patients with clinically probable AD, diagnosed according to the 2011 National Institute on Aging-Alzheimer’s Association criteria, and 40 healthy control participants matched for age and sex. Fasting morning serum concentrations of DHEA-S and cortisol were measured after a 12-hour overnight fast in all participants, and the cortisol/DHEA-S ratio was calculated. Cognitive status was assessed using the Montreal Cognitive Assessment and Clinical Dementia Rating scales.

Results

No statistically significant difference in serum DHEA-S concentrations was found between patients with AD and controls. However, patients with AD had significantly higher cortisol levels (398.85 vs. 337.40 nmol/L; p = 0.026) and a higher cortisol/DHEA-S ratio, showing a trend toward statistical significance (p = 0.078). Among participants aged 65-75 years, the cortisol/DHEA-S ratio was significantly higher in patients with AD than in controls (p = 0.031). In the control group, males had significantly higher DHEA-S levels than females (p = 0.020), whereas no sex difference was observed in the AD group.

Conclusions

The findings of this pilot study suggest that elevated cortisol levels and an imbalance in the cortisol/DHEA-S ratio may contribute to AD pathophysiology. DHEA-S alone did not show a significant association with disease presence, but the observed age- and sex-related differences indicate that this neurosteroid may play a differential role in the development and progression of AD.

## Introduction

Alzheimer’s disease (AD), the most common form of dementia, is one of the leading causes of disability among people over the age of 65 worldwide [1]. It is clinically characterized by a gradual and generalized cognitive dysfunction that most often begins with memory impairment and subsequently affects executive functions, language skills, personality, and behavior [2]. The prevalence of the disease continues to rise, and the number of cases is expected to double within the next 20 years [3].

A major advancement in the understanding and diagnosis of AD occurred in 2011, when the National Institute on Aging-Alzheimer’s Association (NIA-AA) introduced new clinical criteria for probable AD. These criteria incorporated biomarkers to improve diagnostic accuracy while maintaining clinical manifestations as the primary diagnostic standard. Subsequent updates in 2018 and 2024 further refined the diagnostic framework by introducing biomarker-based and biological definitions of AD [4,5].

In the absence of sufficiently effective methods for preventing or curing the disease [2], much research has focused on elucidating its pathophysiology and developing therapeutic strategies aimed at modifying its course. The amyloid hypothesis remains the central framework of modern therapeutic approaches. According to this model, one of the earliest major pathophysiological events is the accumulation of amyloid-β (Aβ) peptides. This initiates a cascade of downstream pathological processes, including intracellular aggregation of phosphorylated tau protein, inflammation, oxidative stress, excitotoxicity, and metabolic dysfunction, ultimately leading to neurodegeneration and the onset of cognitive symptoms. The pathophysiology of AD is complex and involves multiple pathways, such as inflammation, ischemia, impaired energy metabolism, and oxidative damage, that together contribute to progressive neuronal loss [6].

The etiology of the sporadic form of AD involves a complex interaction of genetic predisposition, environmental influences, and epigenetic modifications, which may explain variability in disease onset, rate of progression, and phenotypic expression. The recently proposed probabilistic model of AD does not reject the amyloid hypothesis but recognizes the existence of different disease subtypes with varying contributions of amyloid pathology. This is particularly evident in the sporadic form of AD not associated with the APOE ε4 genotype, where stochastic factors may play a major role in disease onset. This approach emphasizes the need to develop alternative therapeutic targets and individualized prevention strategies [6-8].

Recent research has highlighted the neuroprotective properties of neurosteroids, hormones synthesized de novo in the central nervous system from cholesterol or by the metabolism of peripheral precursors, which may play a role in neurodegenerative diseases such as AD [9,10]. Among these, dehydroepiandrosterone (DHEA) is particularly important, as it is synthesized in neurons and glial cells, predominantly in astrocytes and oligodendrocytes. Brain concentrations of DHEA are six to eight times higher than those in plasma, indicating predominantly local synthesis [11]. Although DHEA is the primary steroid, it is mainly present in circulation as its sulfated derivative, DHEA sulfate (DHEA-S), which can be converted back to active DHEA by steroid sulfatase. Due to its slower elimination, strong binding to serum albumin, and renal tubular reabsorption, plasma DHEA-S levels are relatively stable, making it a reliable biomarker in clinical and research studies [12].

Experimental evidence shows that DHEA and DHEA-S promote neurogenesis and neuronal survival, reduce oxidative stress and inflammation, and mitigate the negative effects of glucocorticoids, particularly cortisol [13,14]. Their antagonistic relationship with cortisol, the key stress hormone, is well documented, with many DHEA/S effects attributed to antiglucocorticoid activity. Chronic stress and systemic diseases may dysregulate the hypothalamic-pituitary-adrenal axis, leading to elevated cortisol and decreased DHEA-S levels, a mechanism potentially contributing to neurodegeneration, especially in older adults [15-19].

DHEA is often described as a “biomarker of aging,” as plasma concentrations peak in the third decade of life and gradually decline thereafter. In individuals over 60 years old, DHEA/S levels are only 10-20% of youthful concentrations. A temporal parallelism has been observed between declining DHEA/S levels and cognitive decline, leading to the hypothesis that reduced levels of these hormones may adversely affect brain function in late life [20].

Animal studies support this hypothesis, showing that DHEA supplementation improves memory and protects hippocampal neurons from oxidative damage. Human studies, however, remain heterogeneous: some report an association between low DHEA/S levels and cognitive decline, while others do not or show opposite trends. Experimental data also suggest sex differences in DHEA/S levels and effects, with higher levels generally observed in men, although population studies remain inconsistent and require further confirmation [14,21-25].

Given that, to our knowledge, similar studies have not yet been conducted in our population, the aim of this study was to examine serum DHEA-S concentrations in patients with AD compared with a control group of healthy, cognitively intact, age- and sex-matched individuals. In addition, this study aimed to assess serum cortisol concentrations and cortisol/DHEA-S ratios in patients with AD compared with controls, as well as to analyze the association of DHEA-S levels with age and sex within both study groups.

## Materials and methods

A clinical observational case-control study was conducted at the Clinic for Neurology, Military Medical Academy (MMA), Belgrade, in accordance with the applicable principles of Good Clinical Practice. The study was approved by the Ethics Committee of the MMA (decision no. 52/2022, dated November 10, 2022) and the Ethics Committee of the Faculty of Medicine, MMA, University of Defense in Belgrade (no. 5719-1, dated December 16, 2022; decision no. 1/3/2022). All participants, patients, and/or their caregivers, as well as cognitively intact control subjects, provided written informed consent to participate in the study after receiving all relevant information.

The study included 45 patients over 60 years of age with a diagnosis of clinically probable AD, established according to the 2011 NIA-AA criteria, and a control group of 40 cognitively intact individuals with no subjective or objective evidence of cognitive impairment.

Exclusion criteria for the patient group included dementias of other etiologies, acute or chronic neurological or psychiatric disorders, head injuries, pronounced vision, hearing, or speech disorders, adrenal gland diseases, severe systemic or malignant diseases, unregulated metabolic disorders, vitamin deficiencies, use of medications that could affect DHEA-S levels (such as corticosteroids, androgens, or estrogens), and the consumption of alcohol or psychoactive substances. For the control group, additional exclusion criteria included any objective evidence of cognitive impairment and a diagnosis of type 1 or type 2 diabetes mellitus.

After inclusion, sociodemographic data, comorbidity information, and lifestyle characteristics were collected using a standardized form that guided a structured interview conducted by a neurologist with all participants and, when applicable, with caregivers of patients with AD. For patients with AD, additional clinical information was obtained from medical records.

In all participants, blood samples were collected in the morning after a 12-hour overnight fast, as previously instructed. Compliance with fasting was verbally confirmed by the phlebotomist before collection. Following blood sampling, clinical examinations were performed. All participants underwent neurological examination and cognitive testing by the same neurologist. Laboratory analyses were conducted at the Institute of Biochemistry, MMA. Basal serum concentrations of DHEA-S and cortisol were determined from venous blood samples drawn from the cubital vein at approximately 8:00 a.m., using the ECLIA method (Cobas E611, Roche, Basel, Switzerland) for DHEA-S and the CLIA method (UniCel DxI 800, Beckman Coulter, Inc., Brea, CA, USA) for cortisol. Based on the measured values, the cortisol/DHEA-S ratio was calculated.

Assessment of cognitive status

Assessment of cognitive status was an integral part of the clinical evaluation for all participants. The Montreal Cognitive Assessment (MoCA) test, which is highly sensitive for detecting mild cognitive impairment, was used. A score of ≥26 points (out of a maximum of 30) demonstrates a sensitivity of 90% and a specificity of 87% for detecting mild cognitive impairment [26]. In the control group, the MoCA test was applied to exclude potential participants with undiagnosed cognitive deficits, thereby ensuring the validity and homogeneity of the sample. In the group of patients with AD, the test was used to descriptively assess the degree of cognitive impairment.

Additionally, patients with AD were evaluated for disease severity using the Clinical Dementia Rating (CDR) scale, in accordance with routine clinical practice. The CDR is one of the most widely used tools for categorizing dementia stages and assessing functional impairment [27].

The results of the MoCA and CDR assessments were used exclusively for the descriptive characterization of the study population and were not included in the correlation analysis with the biochemical parameters, which constituted the primary focus of this study.

Statistical data processing

The sample size was calculated using the formula for determining the difference between the arithmetic means of two groups. The following standard statistical parameters were applied: study power (1-β) = 0.8, significance level (α) = 0.05, and two-tailed testing. The parameters used for the calculation were derived from the study by Aldred and Mecocci [28], in which the mean value of the examined parameter in the experimental group was 2.11 with an SD of 0.42, while the mean value in the control group was 2.46 with an SD of 0.42. For an α level of 0.05 and a study power of 0.8, 24 participants per group (a total of 48) provided 80% power to detect a statistically significant difference of 0.35 (2.11 vs. 2.46, with a common SD of 0.42) in DHEA-S levels using a two-tailed t-test.

Descriptive and analytical statistical methods were employed in this study. Descriptive statistics included absolute and relative frequencies, measures of central tendency (arithmetic mean and median), and measures of dispersion (SD and percentiles). Analytical statistics included difference tests and correlation analyses. For numerical variables, both parametric (t-test) and nonparametric (Mann-Whitney U test and Kruskal-Wallis test) methods were used. The chi-squared test was applied for categorical variables. Pearson and Spearman correlation coefficients were used, depending on data type and distribution. Linear regression analysis was conducted to examine and model the relationship between the dependent variable and one or more independent variables.

The level of statistical significance (type I error probability) was set at p < 0.05. All analyses were performed using IBM SPSS Statistics for Windows, Version 26.0 (Released 2018; IBM Corp., Armonk, NY, USA).

## Results

A total of 45 subjects diagnosed with AD and 40 cognitively intact control subjects were included in the study. There were no statistically significant differences between the groups in terms of age or sex, indicating adequate matching for these demographic parameters. The groups were also comparable with respect to the level of education (Table 1).

**Table 1:** Sociodemographic and clinical characteristics of the study participants ^*^ Chi-square test ^#^ Independent samples test Values are presented as mean ± SD or as an absolute number (percentage). AD, Alzheimer’s disease; CDR, Clinical Dementia Rating; MoCA, Montreal Cognitive Assessment

Variable	Subjects with AD	Control group	p-Value
Gender			
Men	26 (57.8%)	20 (50.0%)	0.617^*^
Women	19 (42.2%)	20 (50.0%)	
Age (years)	76.33 ± 6.66	74.33 ± 7.21	0.185^#^
Education			
1-8 years	3 (6.7%)	3 (7.5%)	0.451^*^
9-12 years	15 (33.3%)	14 (35.0%)	
13-16 years	20 (44.4%)	12 (30.0%)	
>16 years	7 (15.6%)	11 (27.5%)	
MoCA score	17.02 ± 5.85	26.73 ± 1.58	<0.001^*^
CDR score			
0.5	12 (26.7%)	-	-
1	18 (40.0%)	-	-
2	13 (28.9%)	-	-
3	2 (4.4%)	-	-

The MoCA test was administered to both study groups and showed significantly higher scores in the control group. In the control group, the mean score exceeded 26, confirming that none of the participants exhibited symptoms of cognitive impairment. In contrast, the mean MoCA score in the AD group was 17, corresponding to mild cognitive impairment (Table 1).

Regarding the global CDR scale scores among participants with dementia, 26.7% of patients had very mild impairment, 40% had mild dementia, 28.9% had moderate dementia, and 4.4% were in the severe stage (Table 1).

**Figure 1:**
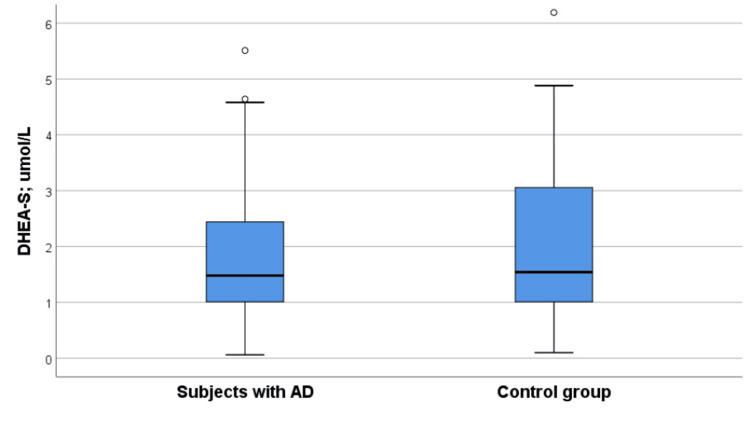
DHEA-S levels in AD and control group subjects AD, Alzheimer’s disease; DHEA-S, dehydroepiandrosterone sulfate

**Figure 2:**
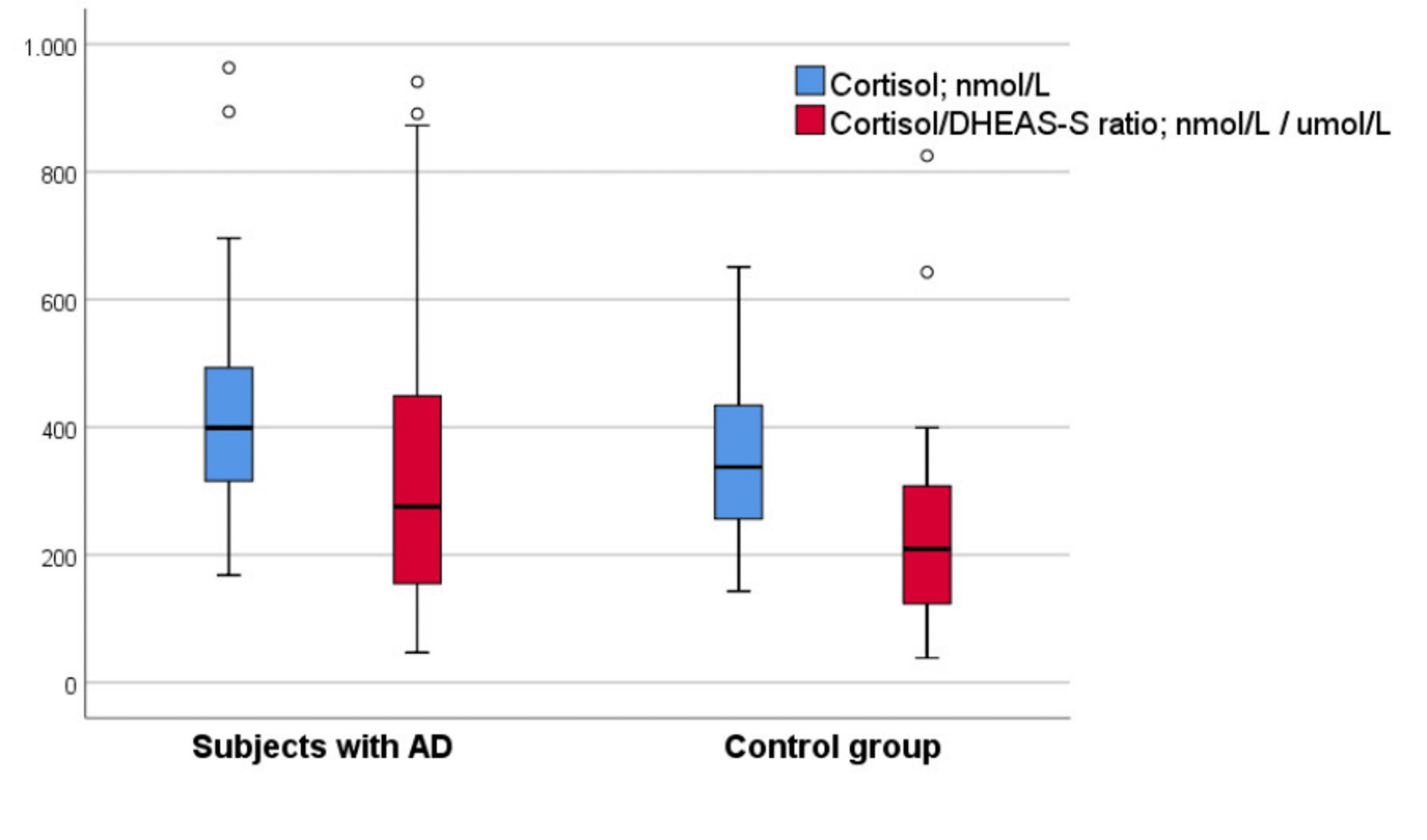
Cortisol levels and cortisol/DHEA-S ratio in AD and control group subjects AD, Alzheimer’s disease; DHEA-S, dehydroepiandrosterone sulfate

Serum DHEA-S levels did not differ significantly between the groups (Figure 1). However, serum cortisol levels were significantly higher in the Alzheimer’s group compared with the control group (median: 398.85 vs. 337.40 nmol/L) (Table 2, Figure 2). The cortisol/DHEA-S ratio was also higher in patients with AD, showing a trend toward statistical significance (Figure 2).

**Table 2:** Laboratory parameters of interest in both examined groups ^*^ Chi-square test ^#^ Mann-Whitney test Values are presented as median with IQR or as an absolute number (percentage). AD, Alzheimer’s disease; DHEA-S, dehydroepiandrosterone sulfate

Variable	Subjects with AD	Control group	p-Value
DHEA-S (µmol/L)	1.48 (0.93-2.47)	1.54 (1.00-3.17)	0.748^#^
Elevated/reduced DHEA-S level	1 (2.2%)/2 (4.4%)	1 (2.5%)/1 (2.5%)	0.887^*^
Cortisol (nmol/L)	398.85 (314.75-493.50)	337.40 (252.00-434.27)	0.026^#^
Elevated/reduced cortisol level	6 (13.6%)/1 (2.3%)	1 (2.9%)/1 (2.9%)	0.260^*^
Cortisol/DHEA-S ratio	275.57 (153.71-463.43)	208.89 (114.03-312.93)	0.078^#^

Analysis by sex showed that, in the control group, men had significantly higher DHEA-S levels than women (median: 2.62 vs. 1.22 µmol/L) (Table 3). In the AD group, no significant differences were observed in DHEA-S levels between men and women. Similarly, no sex-related differences were found in cortisol levels or in the cortisol/DHEA-S ratio in either group.

**Table 3:** Laboratory parameters of interest in both examined groups in relation to gender ^*^ Mann-Whitney test Values are presented as median with IQR. AD, Alzheimer’s disease; DHEA-S, dehydroepiandrosterone sulfate

Variable	Gender	Subjects with AD	Control group	p-Value
DHEA-S (µmol/L)	Male	1.70 (1.05-2.85)	2.62 (1.13-3.75)	0.273^*^
	Female	1.21 (0.53-1.93)	1.22 (0.76-1.73)	0.857^*^
	Sig.	0.141^*^	0.020^*^	
Cortisol (nmol/L)	Male	404.70 (320.45-518.70)	347.90 (237.75-433.97)	0.103^*^
	Female	393.00 (301.00-486.00)	317.25 (259.00-463.50)	0.178^*^
	Sig.	0.767^*^	0.772^*^	
Cortisol/DHEA-S ratio	Male	250.34 (134.67-402.33)	130.75 (65.64-304.05)	0.101^*^
	Female	303.24 (213.56-501.56)	238.00 (173.41-335.30)	0.178^*^
	Sig.	0.110^*^	0.064^*^	

**Figure 3:**
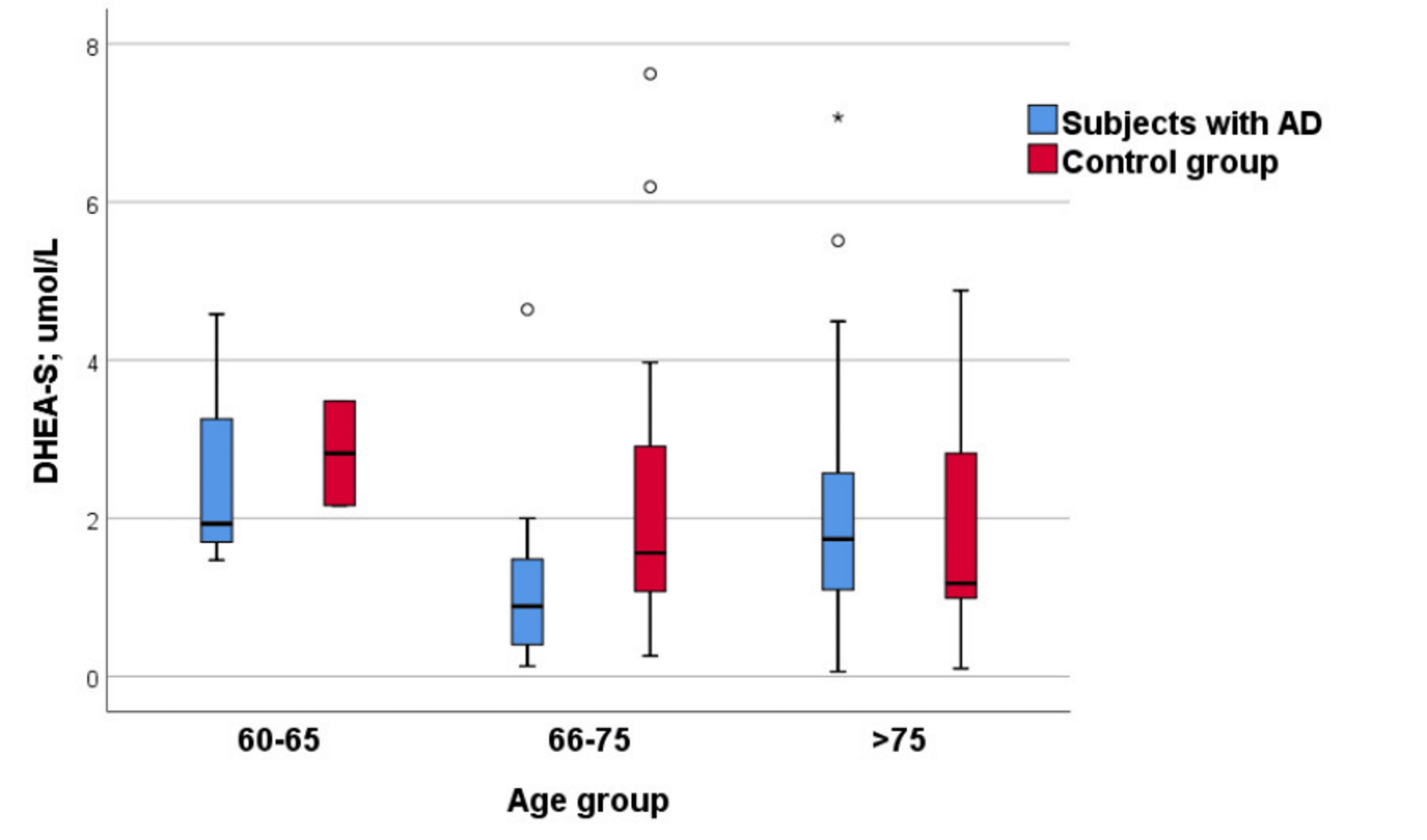
DHEA-S levels in relation to age categories in the AD and control group AD, Alzheimer’s disease; DHEA-S, dehydroepiandrosterone sulfate

When AD patients were stratified by age group, differences in DHEA-S levels were observed: the youngest group showed higher DHEA-S levels than the middle-aged group, followed by an increase again in the oldest group. These differences were close to statistical significance (Kruskal-Wallis test, p = 0.067) (Table 4, Figure 3). In the control group, no statistically significant difference in DHEA-S levels was found among age categories (Kruskal-Wallis test, p = 0.442); however, the youngest subjects tended to have higher DHEA-S levels compared with those in the middle and oldest groups (Figure 3).

**Table 4:** Laboratory parameters of interest in both examined groups in relation to age groups ^*^ Mann-Whitney test ^# ^Kruskal-Wallis test Values are presented as median with IQR. Due to the small size of the subgroup, it was not possible to calculate the upper quartile (Q3) for the 60-65 years age group. AD, Alzheimer’s disease; DHEA-S, dehydroepiandrosterone sulfate

Variable	Age group (years)	Subjects with AD	Control group	p-Value
DHEA-S (µmol/L)	60-65	1.93 (1.47-/)	2.82 (2.16-/)	0.800^*^
	66-75	0.88 (0.38-1.61)	1.56 (1.04-3.10)	0.067^*^
	>75	1.73 (1.06-2.60)	1.17 (0.94-3.02)	0.524^*^
	Sig.	0.067^#^	0.442^#^	
Cortisol (nmol/L)	60-65	305.00 (215.00-/)	306.00 (223.00-/)	0.800^*^
	66-75	336.00 (309.75-455.75)	288.60 (218.50-440.67)	0.226^*^
	>75	411.00 (341.00-561.00)	381.30 (283.25-444.07)	0.186^*^
	Sig.	0.113^#^	0.241^#^	
Cortisol/DHEA-S ratio	60-65	158.03 (46.94-158.03)	122.09 (64.08-/)	1.000^*^
	66-75	355.90 (253.55-835.68)	169.72 (114.03-315.14)	0.031^*^
	>75	259.48 (145.05-404.67)	255.08 (154.67-336.30)	0.607^*^
	Sig.	0.071^#^	0.254^#^	

When comparing the cortisol/DHEA-S ratio between AD and control subjects within each age category, a significant difference was found in the 65-75-year group: patients with AD had a significantly higher ratio than control subjects (medians: 335.90 vs. 169.72) (Table 4).

In the correlation analysis between DHEA-S and other variables of interest, a significant association was found only with sex in the control group: women had lower DHEA-S levels than men (Table 5). No other statistically significant associations between DHEA-S and the examined parameters were observed in any subgroup.

**Table 5:** Correlation of DHEA-S with age and gender ^*^ Spearman correlation AD, Alzheimer’s disease; DHEA-S, dehydroepiandrosterone sulfate

Variable	Subjects with AD^*^	Control group^*^
DHEA-S		
Age	r = 0.063, p = 0.681	r = -0.110, p = 0.497
Gender	r = -0.222, p = 0.143	r = -0.373, p = 0.018
Cortisol		
Age	r = 0.248, p = 0.104	r = 0.234, p = 0.183
Gender	r = -0.045, p = 0.771	r = 0.054, p = 0.761
Cortisol/DHEA-S ratio		
Age	r = -0.005, p = 0.976	r = 0.218, p = 0.216
Gender	r = 0.244, p = 0.111	r = 0.324, p = 0.061

## Discussion

Clinical studies investigating the association between changes in plasma or serum levels of DHEA-S, a neurosteroid, and AD have yielded conflicting results. Few studies have been conducted, and most involved small samples [29-32]. Because of these limited and inconsistent findings, this pilot study aimed to explore differences in the levels of this neurosteroid between patients with AD and healthy control subjects in our population.

In our study, 45 subjects with a diagnosis of probable AD, according to the 2011 NIA-AA criteria [33], were analyzed, while the control group consisted of 40 subjects without signs of cognitive impairment. Among patients with AD, most had mild dementia based on the CDR score. No statistically significant difference was found in serum DHEA-S levels between AD patients and control subjects. This result contradicts several studies that reported lower serum DHEA/DHEA-S levels in AD patients compared with age-matched healthy controls [28,34,35]. A 2019 meta-analysis suggested that a reduced serum concentration of DHEA-S may serve as an important indicator for AD but emphasized that its use as a diagnostic parameter requires further investigation [36]. The same study also found significantly higher DHEA concentrations in AD patients compared with healthy controls in cohorts including individuals older than 80 years. This observation is plausible, as DHEA is a substrate for DHEA-S synthesis, suggesting that the activity of sulfatase and sulfotransferase enzymes, and the molecular mechanisms involved in the bioconversion of DHEA to DHEA-S, may be impaired in AD [36]. It should also be noted that some studies have reported an inverse correlation between AD and serum DHEA-S [37].

Conflicting results among studies conducted in different ethnic populations may be partly explained by socioeconomic, technical, and cultural factors, which have not yet been systematically examined [36]. The significance of our findings lies in providing preliminary data on DHEA-S levels in a Serbian cohort of AD patients compared with cognitively intact controls, a population largely underrepresented in research to date. The findings of this pilot study may reflect the small sample size; however, it is important to note that only a few participants had severe AD, who might have had the greatest influence on group differences. Some previous studies have also reported findings consistent with ours [14]. For example, an Italian study from 2006 found no significant association between blood DHEA-S levels and AD, nor differences between patients and controls after age stratification. That study also found no correlation between reduced DHEA-S levels and specific cognitive domains such as attention and memory, nor with cumulative survival rates in either group [38].

Our study observed significantly higher cortisol levels in patients with dementia, consistent with previous research showing elevated concentrations of this hormone in individuals with AD [16,18,19,39-42]. Cortisol, known as the “stress hormone,” exerts pro-inflammatory and neurotoxic effects at elevated concentrations and contributes to oxidative stress. It is linked to the disruption of Aβ and tau (p-tau) metabolism, which increases the toxicity of these molecules and accelerates neurodegenerative progression. Cortisol also negatively affects synaptic function and reduces dendritic plasticity, particularly in the hippocampus and prefrontal cortex [16]. Elevated cortisol levels have been associated with faster cognitive decline in both healthy older adults and patients with mild cognitive impairment or AD-type dementia [43-46].

Conversely, the neurosteroid DHEA is involved in the metabolism of amyloid precursor protein, whose dysfunction leads to Aβ plaque formation, a key mechanism in AD pathogenesis. DHEA exhibits neuroprotective effects by inhibiting Aβ toxicity via σ1-receptors, exerting anti-inflammatory actions, and modulating glucose and insulin metabolism, which are important in AD [10,47,48]. Postmortem analyses have shown elevated DHEA levels in the prefrontal cortex and hippocampus, possibly reflecting a compensatory response to Aβ accumulation and oxidative stress [49]. Furthermore, the negative correlation between tau protein and DHEA-S in the hypothalamus suggests a possible neuroprotective role for DHEA-S [50].

DHEA and DHEA-S modulate neurotransmitter activity, primarily through acetylcholine, and act indirectly via the gamma-aminobutyric acid (GABA) system as GABA-A receptor antagonists [51,52]. Their antioxidant, antiapoptotic, antiglucocorticoid, and anti-inflammatory properties contribute to neuroprotection. DHEA treatment has been shown to reduce pro-inflammatory cytokines and improve cellular glucose uptake [53]. Because AD is often referred to as “type 3 diabetes” due to its association with insulin resistance and IGF gene abnormalities, the ability of DHEA/DHEA-S to enhance insulin sensitivity and secretion may help reduce the risk of dementia [53,54].

Given the antagonistic relationship between cortisol and DHEA-S, elevated cortisol levels in the early stages of AD may be partially compensated by preserved physiological levels of DHEA-S, which exerts neuroprotective and antiglucocorticoid effects. This response may represent an adaptive mechanism to counteract stress induced by neurodegeneration [55,56]. Therefore, our finding of elevated cortisol levels with preserved DHEA-S concentrations in AD patients could reflect an attempt by the body to restore hormonal balance and mitigate the harmful consequences of glucocorticoid dysfunction [57].

An imbalance in the cortisol/DHEA-S ratio may play a key role in AD pathophysiology. This ratio is considered a more sensitive indicator of the imbalance between chronic stress and neuroprotection, as well as a potential marker of neurodegeneration risk, than individual hormone levels [25]. Our study found that AD patients had a higher cortisol/DHEA-S index compared with controls, approaching statistical significance. Moreover, this ratio was significantly higher in the 65-75-year subgroup of AD patients compared with controls, which was also the largest age group. This finding aligns with previous research demonstrating a significant increase in this index among AD patients [19,31,58]. An imbalance in this hormonal relationship may reflect a transition from a compensated to a decompensated disease phase, serving as an indicator of progression and cognitive decline severity. A recent study showed that elevated CSF cortisol, but not DHEA/DHEA-S, was associated with poorer general cognitive function and more severe disease at baseline. Increased CSF cortisol and cortisol/DHEA-S ratios have also been positively correlated with p-tau levels and negatively associated with amygdala and insula volumes [47,59].

We also observed a significant correlation between sex and DHEA-S levels in the control group, where women had lower values than men, consistent with previous findings [60,61]. Differences in DHEA-S levels between sexes are attributed to biological factors, as men generally exhibit higher concentrations of this hormone, especially at younger ages (25-30 years) [20]. However, this pattern was not observed in the AD group, suggesting that the disease itself may influence hormone regulation. An intriguing question is whether AD leads to a more pronounced reduction in DHEA-S levels among men, thereby eliminating the usual sex-based difference, a hypothesis that warrants further investigation.

Stratification of AD patients by age revealed differences in serum DHEA-S levels: the youngest group (60-65 years) had higher DHEA-S levels than the middle-aged group (66-75 years), while levels were again elevated in the oldest group (>75 years). Although these differences did not reach statistical significance, likely due to the limited sample size, an interesting trend emerged, suggesting possible nonlinear dynamics of DHEA-S across age groups in AD. This finding contrasts with expectations, as DHEA-S typically declines with age due to the physiological reduction of adrenal androgens over time [20,60,62,63]. The increase observed in the oldest group could be influenced by medications commonly used in old age or by compensatory mechanisms that respond to neurodegenerative processes in AD. Hyperlipoproteinemia, which increases with age, might also contribute by elevating substrates for DHEA-S synthesis, though this is less likely. The small sample size and unequal group distribution may also have influenced these observations.

In the control group, DHEA-S levels declined with age, although this trend was not statistically significant, indicating that age-related dynamics differ between AD and non-AD subjects. The observed pattern in AD patients may suggest that the disease exerts a stronger influence on DHEA-S decline than aging itself, particularly if more individuals with moderate AD were included in the middle-aged group. The reduction in DHEA-S levels may thus reflect a decompensatory process in the pathophysiology of moderate AD. This could explain why DHEA-S regulation remains relatively preserved during mild, compensated stages of the disease but becomes impaired as it progresses to moderate stages, resulting in more pronounced hormonal disturbances. The failure of this compensatory mechanism may contribute to the transition toward more severe clinical forms of AD.

It is important to note that this was a pilot study with a limited sample size, which represents a major limitation. Therefore, the findings should be interpreted with caution and confirmed in larger, prospective studies to better clarify these relationships. Future research should include a greater number of patients across all disease stages, particularly severe AD, to improve understanding of hormonal changes throughout disease progression.

## Conclusions

The neurosteroids DHEA and DHEA-S may play an important role in the pathophysiology of AD. The results of our study partially support the hypothesis that lower serum concentrations of DHEA-S could be associated with the development of AD. In this context, the cortisol/DHEA-S ratio also appears to correlate with AD, a finding that was partially confirmed in our study. Elevated serum cortisol levels may induce an increase in DHEA-S as a compensatory mechanism, which could explain the inconsistent results reported in some studies. Assessing neurosteroid levels both peripherally and in the CSF, as well as analyzing their interrelationships, may provide valuable insights for evaluating the risk, progression, and outcome of AD.

Given these findings, there is a need for longitudinal studies to monitor changes in DHEA-S and cortisol levels, as well as their ratio, in relation to AD pathology biomarkers and neuroinflammatory markers. Modulation of neurosteroid activity and levels should also be explored as a potential therapeutic target to prevent and alleviate symptoms and slow disease progression. Larger studies are necessary to establish reference DHEA-S values for our population, as genetic, environmental, and lifestyle factors may influence biochemical marker variations among different ethnic groups.
